# Nasolaryngoscopy or Laryngotracheoscopy: Which is the best exam for assessing the airways of children?

**DOI:** 10.1016/S1808-8694(15)30994-0

**Published:** 2015-10-19

**Authors:** Clarissa Luciana Buono Lehoczki, Daniela Carvalho, Ronny Tah Yen Ng, Reinaldo Jordão Gusmão

**Affiliations:** a SBORL specialist (2004), physician, otorhinolaryngologist, collaborator of the Pediatric Otorhinolaryngology Unit at UNICAMP; b Fellowship in Pediatric Otorhinolaryngology done at the Children's Hospital San Diego UCSD CA/USA, Physician at the Pediatric Otorhinolaryngology Unit at the Children's Hospital San Diego CA/USA; c Otorhinolaryngology Resident - 3rd year at UNICAMP; d Doctor in Otorhinolaryngology at FCM-UNICAMP, Head of the Pediatric Otorhinolaryngology Unit at UNICAMP - Campinas State University

**Keywords:** endoscopy, laryngotracheoscopy, nasolaryngoscopy

## Abstract

It is not uncommon to find children with respiratory distress. In these cases airway endoscopy is usually required. Doubts about which examination should be used are frequent.

**Aim:**

to establish which examination is the best to assess the airways of children.

**Material and Methods:**

a retrospective study assessing 16 children with a history of respiratory distress at the Children Airway Unit of the Pediatric Otolaryngology Department at UNICAMP. All patients underwent nasolaryngoscopy and laryngotracheoscopy between March 2001 and March 2004. Data was analyzed and compared.

**Results:**

during this study 16 children were assessed; the most frequent indication of exams were: evaluation of prolonged tracheostomy in 10 patients (62%), and subglottic stenosis (31.3%).

**Conclusion:**

assessing airways in children with respiratory distress is essential for a diagnosis. In our study, we concluded that all children with upper airway disease must undergo nasolaryngoscopy, an easy, economic and useful exam that provides information about larynx function. However, if subglottic or traqueal disease issuspected, or if nasolaryngoscopy findings are in conflict with the physical examination, laryngotracheoscopy should be undertaken.

## INTRODUCTION

Airway endoscopy has historically been used as an important diagnostic tool to assess children with stridor or other signs of respiratory distress. Technological developments in modern equipment together with safer anesthesia techniques have led to the frequent use of airway endoscopy devices even in the newborn^1^, ^2^.

Airway endoscopy provides the otorhinolaryngologist with a direct view of the affected anatomical site, allowing the diagnosis and in some cases, the treatment of medical conditions.

Two exams should be remembered when speaking of airway visualization. Nasolaryngoscopy (NL), the first of them, is a frequently done exam that does not require sedation, and may be conducted in the medical office with no more than child restraint. The second exam is laryngotracheoscopy (LTQ), which requires general anesthesia, implying higher cost and increased risk.

There are undoubted benefits and disadvantages to justify both exams. Benefits of NL include the ease of the procedure and the possibility of dynamic and functional airway assessment. The advantage of LTQ is the possibility of complete airway visualization down to the subglottis and trachea, together with improved image quality and definition.

Disadvantages of NL include the fact that good assessment of the subglottis is only possible in 30% of children and that the trachea cannot be evaluated; this exam also has a lower image quality. Disadvantages of LT include its higher cost due to the need for general anesthesia, and the fact that it is a static exam, not allowing the evaluation of laryngeal movement and coordination.

Recent debates on which would be best exam to assess child airways have been published in literature^2^, ^4^, ^7^, ^9^.

The most frequent indications for these exams, according to published data, are stridor, progressive respiratory obstruction, feeding difficulties, and when an image diagnosis suggests airways alterations^2^, ^3^, ^4^.

There are groups that defend the use of both exams in all children with stridor or respiratory distress. The justification is that there are synchronic lesions in 30 to 70% of cases, depending on the study. Other groups recommend initial NL followed by LT depending on results and the clinical picture^4^, ^6^.

## OBJETIVE

To compare the advantages and disadvantages of two types of endoscopy (laryngotracheoscopy and nasolaryngoscopy) used to assess the airways of children.

## MATERIAL AND METHODS

We conducted a retrospective review of the charts of children monitored at the Pediatric Otorhinolaryngology Unit outpatient child airway disease clinic at the UNICAMP (Campinas State University) Clinical Hospital between March 2001 and March 2004.

All children up to 14 years of age that had undergone laryngotracheoscopy and nasolaryngoscopy were included in this study. Children in which only one of these exams was done were excluded.

For LT, the patient was taken to the surgical theater and given general anesthesia, followed by the placement of an child Parsons laryngoscope. The upper dental arch was protected with moist gauze. Lidocaine 2% was sprayed over the vocal folds before beginning the exam to avoid laryngospasm and to reduce the need for anesthetic gas. Endoscopy was done using a Hopkins II (Storz®) 0 degree, 2.9 or 4.0 mm diameter, 36 or 30cm length endoscope, respectively.

For NL vasoconstriction was obtained with topical oxymetazoline; for patients aged over 1 year with no neurological compromise, we used topical neotutocaine for local anesthesia. The exam was done using a Machida 3.5mm nasolaryngoscope.

Data such as age, gender, findings and complications of each exam were collected and compared.

## RESULTS

During the three-year period of this review, 38 children underwent laryngotracheoscopy at the UNICAMP Otorhinolaryngology and Head & Neck Unit. However, only 16 underwent ambulatory nasolaryngoscopy, and these were the patients included in this study. Of these children, 11 were male (68.8%) and 3 (31.2%) were female. Age varied from 2 to 156 months, with an average 53 months (four years and four months) at the time of the exam.

**Figure 1:**
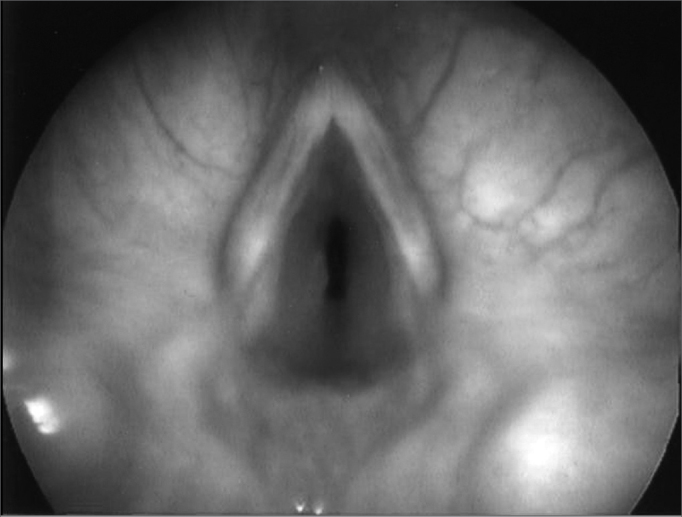
Subglottic edema.

**Figure 2:**
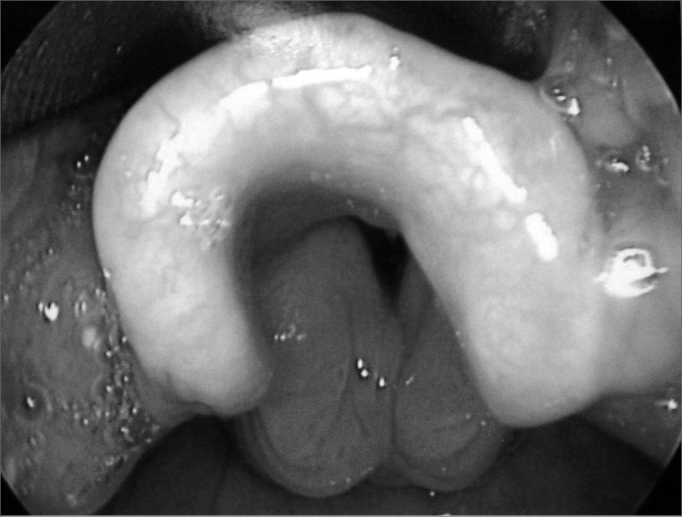
Edema in the interarytenoid region.

The most common indication for the exam was the need to assess prolonged tracheotomy in 10 patients (62%), followed by the assessment of subglottic stenosis in 3 cases (31.3%) (Figure 1). Other indications responded for 31.3% of cases, including dyspnea, laryngitis, the evaluation of gastroesophageal reflux (Figure 2) and extubation difficulties. There was more than one indication for the exam in 7 children (43.8%) (see Table 1).

**Table 1:** Results of laryngotracheoscopy and nasofibroscopy (S/a = no change; ESG = subglottic stenosis; IA = intrarytenoid; N/e = not examined; PPVV = vocal folds.)

Patient	Laryngotracheoscopy				Nasofibroscopy			
	Supraglottis	Glottis	Subglottis	Trachea	Supraglottis	Glottis	Subglottis	Trachea
1	S/a	Edema	ESG	Granuloma	Hyperemia	S/a	ESG	N/e
2	Edema	Edema	ESG	Secretion	Edema IA	S/a	ESG	N/e
3	S/a	S/a	S/a	Bulging	S/a	S/a	S/a	N/e
4	Edema	S/a	S/a	Bulging	Edema Ia	S/a	S/a	N/e
5	Edema	S/a	ESG	Collapse	Edema Ia	S/a	S/a	N/e
6	Edema	Edema	ESG	Not visible	S/a	S/a	ESG	N/e
7	S/a	Nodules	S/a	Collapse	S/a	S/a	ESG	N/e
8	S/a	S/a	ESG	S/a	S/a	S/a	ESG	N/e
9	Arytenoid fixation	Synechia	ESG	Stenosis	S/a	S/a	ESG	N/e
10	Edema	S/a	ESG	S/a	Hyperemia IA	S/a	S/a	N/e
11	S/a	S/a	ESG	S/a	S/a	Granuloma PPVV	ESG	N/e
12	Edema	Fibrin	Edema	Granuloma	S/a	S/a	S/a	N/e
13	S/a	Granuloma	ESG	S/a	S/a	S/a	ESG	N/e
14	Edema	S/a	ESG	S/a	S/a	S/a	S/a	N/e
15	S/a	S/a	ESG	S/a	S/a	S/a	ESG	N/e
16	Edema	S/a	Edema	Granuloma	S/a	S/a	S/a	N/e

## DISCUSSION

Respiratory distress is a relatively common problem in children, and there is no clinical protocol to be followed. In this case we should ask ourselves how far to go in making the diagnosis and conducting the case. Most children with respiratory distress present laryngomalacia^1^, ^2^. Our study had different finding, as most children were diagnosed based on the clinical history, the physical exam and nasolaryngoscopy. Even in children with laryngomalacia there are concomitant findings named synchronous lesions reported in literature in 8 to 45% of cases^2^, ^3^, ^4^. The fear of missing synchronous lesions led to the debate on whether to perform routine LTQ in every children presenting with respiratory distress or discomfort. Undoubtedly rigid LTQ provides added information about the glottis and the trachea; this exam, however, requires general anesthesia, which increases cost and requires time in a surgical theater. Taking these issues into account, the question was when to do this exam?

NL is a useful tool as a first line exam when investigating respiratory distress^4^, ^8^. It is a safe procedure, allowing good visualization of laryngeal dynamics; however, visualization of the subglottis is only possible in 25% of cases^4^. In our study failure to see subglottic alterations using NL occurred in 44% of exams. Possible complications include laryngospasm, epistaxis and aspiration. No complication occurred in our study, possible due to the fact that we did not use sedation and did not proceed beyond the vocal folds.

Even if synchronous lesions exist in these children, they will become evident on follow-up if significant.

Some authors recommend doing LT together with NL in every child with respiratory distress. However, according to our findings and those by other authors^2^, ^3^, ^4^, a two-step approach, with initial NL followed by LTQ if the diagnosis is not clear, would be preferable.

Success in doing only NL initially requires certain measures. First of all, the child needs to have developed adequately, eating and gaining weight normally. Secondly, the type of stridor has to be taken into account; children with biphasic or expiratory stridor, which speaks in favor of subglottic or tracheal disease, should necessarily undergo LTQ for complete airway evaluation, even if there are alterations seen on NL. In these cases, the physical exam will not match NL findings.

Using only NL initially does not mean that LTQ will not be used later. It means saving the parents’ time, avoiding the use of endovenous catheters, reducing the risk of sedatives and anesthetic drugs, avoiding, fever, infection, and reducing cost^2^.

Parents should always be made aware of this strategy and should know that if there are changes in the respiratory pattern with worsening of the clinical picture, or changes in height or weight gains, they should bring the child for medical reassessment and possible LTQ.

**Figure 3:**
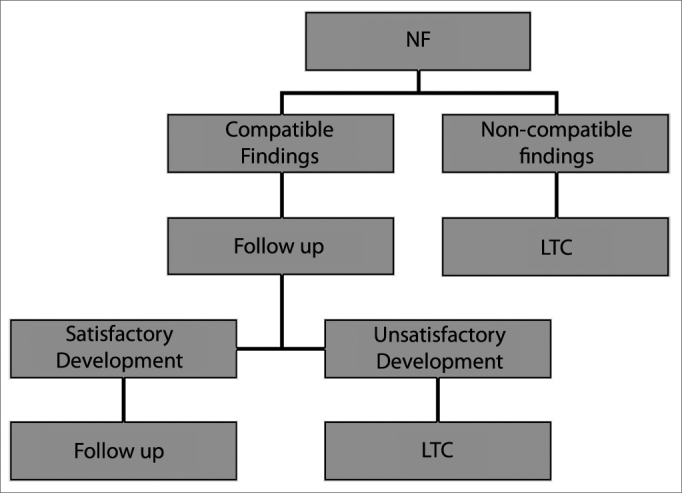
Assessment of the airways of children NF = nasofibropharyngoscopy; LTQ = laryngotracheoscopy.

It is clear that our sample contained a high incidence of subglottic stenosis (Figure 2) and that in some cases this diagnosis was not made on NL, as expected. However, other findings disclosed on LTQ and not seen on NL would not in themselves be sufficient to change the clinical progression of these children. We therefore suggest a child airway assessment flowchart (Figure 3) to guide the clinical investigation of these patients.

## CONCLUSIONS

We conclude that NL is a simple, safe, and easy exam that provides important information to diagnose airway diseases, and for this reason should always be used as a first choice diagnostic tool. LT is also a useful exam, particularly when subglottic disease is suspected; however, it incurs in greater cost and added risk, and although it provides relevant information, it should be left for cases where subglottic disease is suspected or when the patient's symptoms are not explained by NL findings.
